# Circulating Differentially Methylated Amylin DNA as a Biomarker of β-Cell Loss in Type 1 Diabetes

**DOI:** 10.1371/journal.pone.0152662

**Published:** 2016-04-25

**Authors:** John A. Olsen, Lauren A. Kenna, Michael G. Spelios, Martin J. Hessner, Eitan M. Akirav

**Affiliations:** 1 Research Institute, Islet Biology, Winthrop-University Hospital, Mineola, NY, United States of America; 2 Max McGee National Research Center for Juvenile Diabetes, Children's Research Institute of Children's Hospital of Wisconsin, Milwaukee, WI, United States of America; 3 Department of Pediatrics, Medical College of Wisconsin, Milwaukee, WI, United States of America; 4 Stony Brook University School of Medicine, Stony Brook, NY, United States of America; Joslin Diabetes Center, Harvard Medical School, UNITED STATES

## Abstract

In type 1 diabetes (T1D), β-cell loss is silent during disease progression. Methylation-sensitive quantitative real-time PCR (qPCR) of β-cell-derived DNA in the blood can serve as a biomarker of β-cell death in T1D. Amylin is highly expressed by β-cells in the islet. Here we examined whether demethylated circulating free amylin DNA (cfDNA) may serve as a biomarker of β-cell death in T1D. β cells showed unique methylation patterns within the amylin coding region that were not observed with other tissues. The design and use of methylation-specific primers yielded a strong signal for demethylated amylin in purified DNA from murine islets when compared with other tissues. Similarly, methylation-specific primers detected high levels of demethylated amylin DNA in human islets and enriched human β-cells. In vivo testing of the primers revealed an increase in demethylated amylin cfDNA in sera of non-obese diabetic (NOD) mice during T1D progression and following the development of hyperglycemia. This increase in amylin cfDNA did not mirror the increase in insulin cfDNA, suggesting that amylin cfDNA may detect β-cell loss in serum samples where insulin cfDNA is undetected. Finally, purified cfDNA from recent onset T1D patients yielded a high signal for demethylated amylin cfDNA when compared with matched healthy controls. These findings support the use of demethylated amylin cfDNA for detection of β-cell-derived DNA. When utilized in conjunction with insulin, this latest assay provides a comprehensive multi-gene approach for the detection of β-cell loss.

## Introduction

In type 1 diabetes (T1D), β-cell loss is a silent process which leads to the development of hyperglycemia. The inability to detect β-cell loss limits early disease diagnosis, prognosis, and the opportunity for intervention prior to clinical onset. Although several biomarkers of immune activation and β-cell function can be used to evaluate the risk of developing T1D, these biomarkers are limited in their ability to detect active β-cell loss [1].

DNA methylation represents a native process whereby tissue-specific genes are regulated [2]. In general, DNA hypermethylation is associated with gene silencing and suppression, while DNA demethylation correlates with increased expression. Insulin expression in β-cells is mediated in part by altered DNA methylation. For example, insulin promoter hypomethylation of CpG dinucleotides is detected in insulin-positive β-cells, while absent in other tissues [3]. These differential methylation patterns can be detected by bisulfite DNA conversion followed by methylation-specific quantitative real-time PCR (qPCR) [4].

Circulating free DNA (cfDNA) can be used for the detection of remote cell loss. For example, in cancer cfDNA is used as a “liquid biopsy” for the detection of tumor growth based on previously documented DNA mutations and epigenetic modifications [5–7]. Our laboratory and others have previously showed the utility of differentially methylated insulin DNA as a biomarker of β-cell loss in patients and animals with T1D [8–12]. Examination of β-cell derived *insulin* cfDNA levels revealed an increase in total β-cell DNA in serum of the non-obese diabetic (NOD) mouse model of T1D and in patients with recent onset type 1 diabetes [8, 10].

Islet Amyloid Polypeptide (IAPP), also known as amylin, is a gene expressed predominantly in β-cells [13, 14]. Amylin is co-secreted with insulin from the secretory granules [15–17], and shares similar transcription elements with the insulin gene [18–20]. The amylin peptide is 37 amino acids in length, and has been identified as the primary component of amyloid deposits observed in the islets of type 2 diabetes (T2D) patients [21–23]. Amylin secretion has been linked to satiety and inhibition of glucagon secretion [24–26]. Current therapy for T1D and T2D includes the use of amylin analogs for controlling body weight and lowering blood glucose levels [27–30]. The specific expression of amylin in β-cells suggests that gene expression may be regulated by methylation, making it a viable candidate for use with our assay in the detection of β-cell death.

In this report, we show differential methylation of the amylin gene in insulinoma cells and primary islets of murine origin, suggesting that amylin demethylation can be used as a biomarker of β-cell loss in circulation. Methylation-specific amylin primers show the ability to detect increased β-cell death in the NOD mouse model of T1D. Examination of amylin expression in the islet during T1D progression reveals a disconnect from insulin expression during the late stages of the disease, suggesting that amylin may be used to detect an insulin-negative β-cell fraction that would otherwise go undetected by an insulin-based biomarker assay. In T1D patients, amylin cfDNA is increased following disease onset demonstrating the utility of this biomarker in human disease.

## Materials and Methods

### Mice

Female NOD/LtJ mice were obtained pathogen-free from the Jackson Laboratory (Bar Harbor, ME) and maintained under pathogen-free conditions. Eight-wk old NOD mice were screened for hyperglycemia every 2–4 wks and were diagnosed with diabetes when glucose levels >200 mg/dL were measured in whole blood from the tail vein using a Glucometer Elite XL (Bayer A.G., Whippany, NJ). Blood for demethylation index (DMI) analysis was collected by cheek pouch bleeding, thereby allowing for monitoring of β-cell death in the same animal until the development of frank hyperglycemia. All animal useand husbandry protocols were approved by the Winthrop-University Hospital Institutional Animal Care and Use Committee.

### IPGTT

Intraperitoneal glucose tolerance test (IPGTT) was done as previously described [31]. In brief, mice undergoing an IPGTT were fasted overnight and received a 2 g/kg intraperitoneal (i.p.) dextrose injection. Whole-blood glucose levels were measured from the tail vein at 0 15, 30, 60, and120 min after injection.

### Immunofluorescence

Immunofluorescence was done as previously described [31]. Pancreata were resected and fixed for 24 h in 2% PFA. After fixation, pancreatic tissues were placed in a sucrose gradient and snap frozen in liquid nitrogen. Noncontiguous 14-mm pancreatic sections were stained with antibodies to insulin (Abcam, Cambridge, MA), amylin (Abcam, Cambridge, MA), and glucose transporter 2 (GLUT2, Santa Cruz, Santa Cruz, CA). The bound antibodies were detected by immunofluorescent secondary antibodies (Jackson Immunoresearch, West Grove, PA). Nuclear staining was done using 4’,6-diamidino-2-phenylindole dihydrochloride (DAPI), The slides were analyzed by fluorescence microscopy using a Nikon Eclipse Ti confocal microscope (Nikon, Melville, NY).

### Cell Lines

MS1 mouse pancreatic islet endothelial cells (American Type Culture Collection, Manassas, VA, catalog number CRL-2279) were cultured and stored using provided protocols. Mouse βTC3 insulinoma cells were a gift from Albert Einstein College of Medicine (Bronx, NY), and culture protocols are previously described in [32, 33]. Human EndoC-βH1 cells were obtained from Dr. R. Scharfmann laboratory, (CRICM, Paris, France) and were cultured as previously described ([34].

### Human Islets

Islet samples were received from the Integrated Islet Distribution Program (IIDP, Duarte, CA) (donor numbers 971, 1265 and 1393).

### Human β-Cell Fractions

Primary β-cells were isolated by magnetic bead purification using the AutoMACS cell sorter (Miltenyi Biotech Inc., San Diego, CA) as previously described [35]. In brief, human islets were washed once, subjected to trypsin disassociation, and filtered using a 70 μm nylon mesh. Dispersed islets were stained using anti-human CA19-9 antibodies (Miltenyi Biotech Inc., San Diego, CA) and ran through the AutoMACS. The negative fraction was stained using PSA-NCAM microbeads (Miltenyi Biotech Inc., San Diego, CA) and the positive fraction was sorted using the AutoMACS. CA19-9^-^PSA-NCAM^+^ cells were enriched for β-cells and were used for further analysis. Fraction enrichment was verified by measuring the signal of demethylated insulin DNA as previously described [8] (S1 Fig).

### Research Subjects

Recent onset (RO) T1D and unrelated healthy control (HC) subjects were recruited through Children’s Hospital of Wisconsin (CHW). RO T1D subjects (n = 15) met diagnostic criteria of T1D as defined per World Health Organization criteria [36] and were positive for >1 AA. Samples were collected 2–7 months after clinical onset from subjects with histories of good glycemic control. HC (n = 11) were free of known infection at sample collection and did not possess a family history of T1D. Details of the studied subjects are provided in Table 1. The study was approved by the Institutional Review Board of CHW (IRB 01–15) and written informed consent was obtained from subjects or their parents/legal guardians.

**Table 1:** Patient demographics and clinical information.

Parameters	Groups	
	Ctrl	T1D
**N**	11	15
**Age**	13.13±1.00	13.22±066
**F/(M)**	5/(6)	8/(7)
**Age at Dx**	-	12.90±0.68
**HbA1c**	-	7.5±0.28%
**# AutoAb**	0	3.18±0.26

### DNA Extraction and Bisulfite Treatment

DNA was purified from sera, pelleted cells, and homogenized tissue using the DNEasy Blood and Tissue Kit (Qiagen N.V., Valencia, CA). The concentration of the purified DNA was measured using the Quant-iT PicoGreen dsDNA Assay Kit (Life Technologies, Carlsbad, CA). Purified DNA was bisulfite treated using the EZ DNA Methylation-Direct Kit (Zymo Research, Irvine, CA).

### First-Step PCR and Gel Extraction

**Fig 1:**
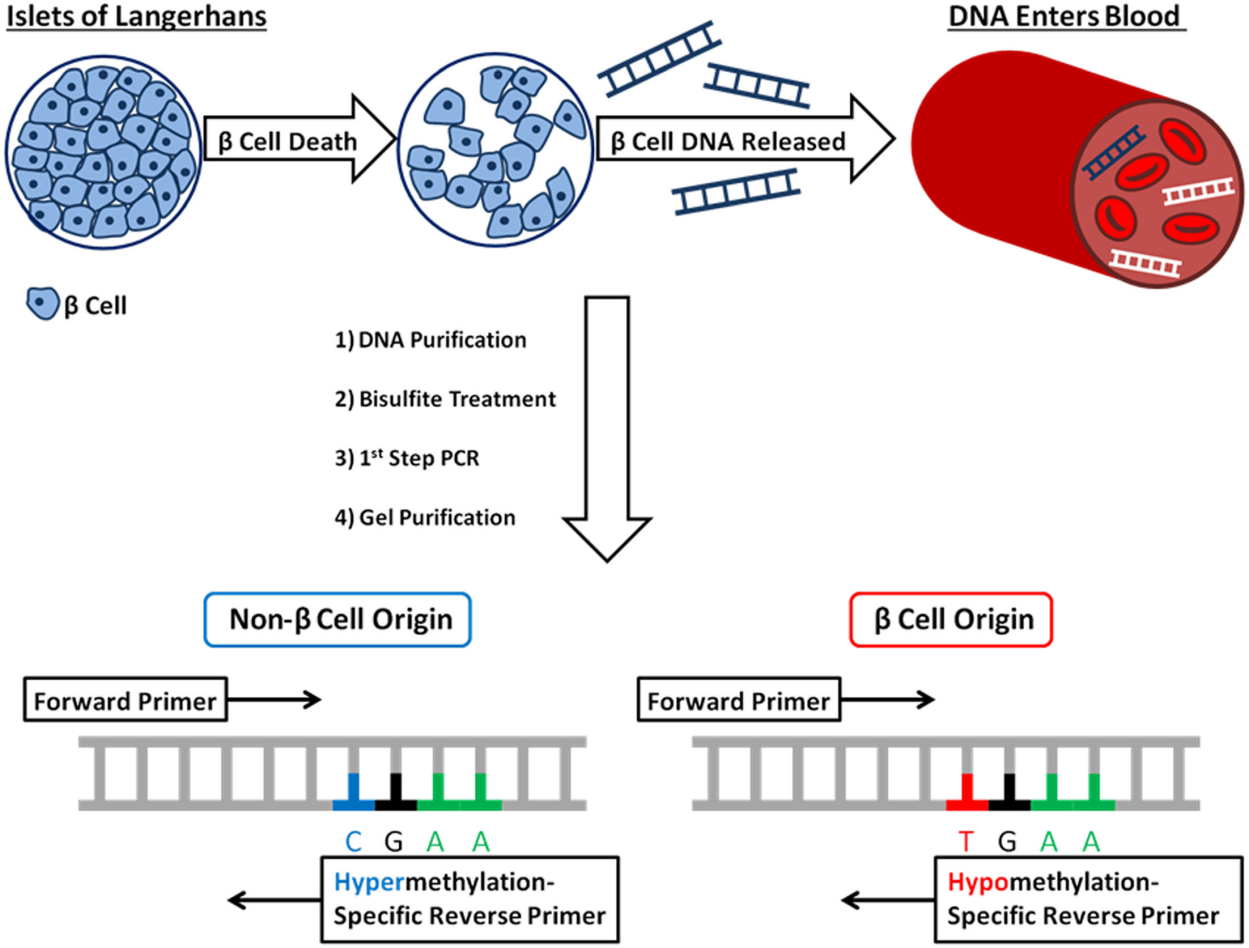
A schematic depiction of amylin-based biomarker assay for the detection of β-cell loss in T1D. β-cells within the islets of Langerhans die, releasing genomic DNA into circulation. Blood samples are taken from subject and DNA is purified and subjected bisulfite conversion. Bisulfite converted DNA is subjected to first step PCR reaction using methylation unspecific primers, and run on agarose gel. First step PCR product is purified from agarose gel and used as template for qPCR using methylation-specific primers.

Prior to qPCR analysis, a non-methylation-specific PCR was run in order to increase DNA template. Sequences for the human and murine primers can be found in Tables 2 and 3. Bisulfite treated DNA was used as template for the reaction which was run using the EpiTaq HS Kit (Clonetech Laboratories Inc., Mountain View, CA). PCR protocols for both murine and human reactions are listed in Tables 2 and 3. PCR products were run on a 2% agarose gel and purified using the QIAquick Gel Extraction Kit (Qiagen N.V., Valencia, CA) (Fig 1). No template controls were used to exclude DNA contamination and showed no observable products in the first-step PCR reaction.

**Table 2:** Primer sequences and PCR protocols for mouse amylin analysis.

PCR Type	Primer Designation	Primer Sequence 5’→3’	Product Length	PCR Protocol
**First-step PCR**	Forward	TGGTAGTAATTTTTAGATGGATAAA	178bp	50 Cycles, annealing temperature
	Reverse	AAATTCCCTATTTAAATCCCCTAC		57°C
**Methylation-specific nested qPCR**	Hypermeth -specific forward	AAACGGAAGTGTAATACGGTTAC	122bp	40 Cycles, annealing temperature 63°C
	Hypermeth-specific reverse	TTACCATATATATTCGATCCCACG		
	Hypometh-specific forward	AAATGGAAGTGTAATATGGTTAT		
	Hypometh-specific reverse	TTACCATATATATTCAATCCCACA		

**Table 3:** Primer sequences and PCR protocols for human amylin analysis.

PCR Type	Primer Designation	Primer Sequence 5’→3’	Product Length	PCR Protocol
**First-step PCR**	Forward	TGTTATTAGTTATTAGGTGGAAAAG	146bp	50 Cycles, annealing temperature 57°C
	Reverse	TCTTACCATATATATTAAATCCCAC		
**Methylation-specific nested qPCR**	Common forward	TGTTATTAGTTATTAGGTGGAAAAG	76bp	40 Cycles, annealing temperature 63°C
	Hypermeth-specific reverse	TAAAAAATTTACCAAACGCTACG		
	Hypometh-specific reverse	TAAAAAATTTACCAAACACTACA		
**Native Amylin PCR**	Forward	TGTTACCAGTCATCAGGTGGAAAAG	146bp	27–33 Cycles, annealing temperature 57°C
	Reverse	TCTTGCCATATGTATTGGATCCCAC		

### Cloning Reaction and Sequencing of Amylin DNA

Gel purified human and murine islet DNA samples previously bisulfite treated and run on first-step PCR were used in a TOPO TA Cloning reaction and ligated to pCR 2.1TOPO vector (Invitrogen, Carlsbad, CA). Competent NEB 5-alpha *E*. *coli* (New England Biolabs, Ipswich, MA) were transformed with the TOPO ligation products, plated on ImMedia Kan Agar (Invitrogen, Carlsbad, CA), and incubated at 37°C overnight. Colonies from both human and mouse samples were isolated, added to LB broth/Kanamycin culture, and shaken overnight at 37°C. TOPO plasmid DNA was purified from the cultures using the QIAprep Spin Miniprep Kit (Qiagen N.V., Valencia, CA). Purified plasmid DNA and gel extracted first-step PCR products were sequenced at the Keck Biotechnology Research Laboratory (New Haven, CT).

### McrBC Restriction Enzyme Reaction

Purified DNA from human liver and beta cell fraction was treated with the McrBC methylation-specific restriction enzyme (New England Biolabs Inc., Ipswich, MA). After treatment, 0.8 ng of treated and untreated liver and beta cell fraction DNA, as well as a TOPO plasmid containing the appropriate native amylin insert (625,000 copies per reaction), were run on PCR using native amylin primers (Table 3). Samples run on PCR were removed at cycle 27 or 30. PCR products were run on a 2% agarose gel and imaged using a 4000R Image Station (Eastman Kodak Co., Rochester, NY).

### Nested Methylation Sensitive qPCR

Gel-purified first-step PCR products were used as template for qPCR using primers designed for bisulfite-converted demethylated and methylated amylin DNA. Both murine and human reactions were run using SsoAdvanced Universal SYBR Green Supermix (Bio-Rad Laboratories Inc., Hercules, CA). Primers and PCR protocols are reported in Tables 2 and 3. All reactions were run on a CFX96 Real Time System (Bio-Rad Laboratories Inc., Hercules, CA). Relative quantification of demethylated DNA was calculated by DMI = 2^(methylated cycle number)–(demethylated cycle number)^.

### Statistical Analysis

Results are presented as mean ± SEM. Statistical significance (p < 0.05) of differences between means was determined by one-way ANOVA with Tukey’s post hoc test using Prism 5 (GraphPad software).

## Results

### The amylin gene is differentially methylated in primary islets and murine insulinomas and can be detected by methylation-specific primers

**Fig 2:**
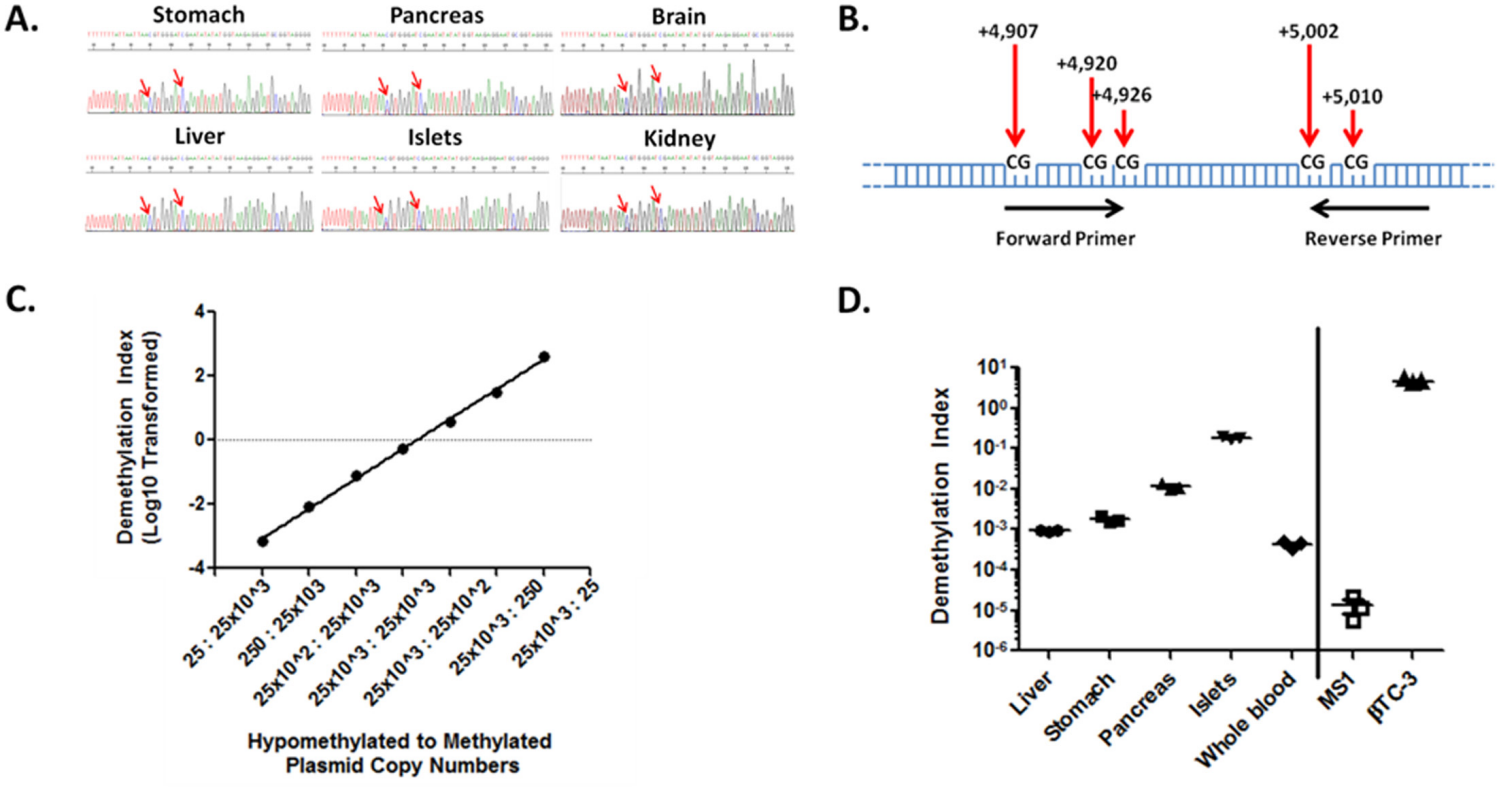
Amylin DNA is demethylated in murine pancreas, islets and β-cells and can be selectively detected using demethylation specific primers. ***A*.** Sanger sequencing of bisulfite treated DNA from various tissues. Red arrows point to a mixed signal consisting of cytosine (C) and thymine (T) in DNA from whole pancreas and purified mouse islets, indicating a mixed population of methylated CpG dinucleotides. ***B*.** Schematic depiction of differentially methylated CpG dinucleotides in the mouse amylin coding region used for the design of methylation-specific primers. Nucleotide position is from transcription start site. ***C*.** Methylation-specific primers were tested over a wide range of copy numbers for DeMeth and Meth DNA (25 to 25x10^3^) to determine assay sensitivity and specificity using cloned DNA sequences. Demethylation index was calculated as (DMI) = 2^(methylated cycle number)–(demethylated cycle number)^. (R^2^ = 0.9981, p<0.0001). ***D*.** qPCR reaction using methylation-sensitive primers on bisulfite treated DNA from liver, stomach, pancreas, islets and whole blood. βTC3 insulinoma (positive control) and the immortalized murine cell line MS1 (islet endothelium—negative control) were used as controls. Data consists of three independent analyses. Assay reproducibility as measured by CV = 11.32 ± 2.68%.

The insulin gene exhibits differential DNA methylation of the insulin promoter and coding sequences in β-cells [3, 8]. Similarly, amylin is expressed predominantly by β-cells and is secreted together with insulin [15–17], therefore suggesting that amylin DNA may be uniquely demethylated in these cells. Sequence analysis of bisulfite-converted DNA from murine brain, kidney, liver, small intestine and stomach revealed a complete methylation of CpG dinucleotides in the coding region of the amylin gene (Fig 2A and data not shown). In contrast, sequence analysis of DNA from murine pancreas and purified islets revealed a mixed population of C/T nucleotides post bisulfite conversion, suggesting that the amylin gene is demethylated in β-cells (Fig 2A).

The reduced DNA methylation in both pancreas and primary islets prompted us to examine whether methylation-specific primers are capable of differentiating methylated (Meth) from demethylated (DeMeth) amylin DNA. Five differentially methylated CpGs were chosen to be included in the forward and reverse primer sequences (Fig 2B and Table 2). Synthetic DNA representing Meth or DeMeth amylin sequences were synthesized, cloned and sequence validated. These plasmids were then used to define primer sensitivity and specificity to their respective templates. Plasmids were mixed at variable copy numbers of 25, 250, 25x10^2^ and 25x10^3^ copies representing a range of 12.5 to 12,500 β-cells, respectively, over a six logarithmic concentration range and used to determine the specificity and sensitivity of methylation-specific primers. The relative abundance of the DeMeth DNA (representative of β-cell DNA) was expressed by using DMI as described in the materials and methods. Fig 2C shows a strong linear correlation between increasing DeMeth DNA concentrations and DMI values (R^2^ = 0.9981, p<0.0001), demonstrating the ability of DeMeth specific primers to detect DeMeth DNA over a wide range of copies. Finally, we examined whether methylation-specific primers detected β-cell DNA in primary tissues and murine cell lines. DMI values for purified mouse islets were ~190 fold higher than those measured in liver, indicating a high level of specificity of the DeMeth primers (Fig 2D). Since DMI values are determined in cell-free serum, we examined the DMI levels of amylin in whole blood preparations from healthy mice. DMI values from whole blood showed low DMI levels similar to those detected in the liver and were significantly lower than the levels detected in purified islets (Fig 2D). Comparison of DMI values between the murine insulinoma line, βTC3, and the islet-derived endothelial cell-line MS1 showed a ~10^6^ increase in the former (Fig 2D). Taken together, these data identify previously unreported demethylated CpG dinucleotides in the coding region of the amylin gene in primary islets and in βTC3 insulinomas. Primers designed to differentiate between Meth and DeMeth amylin DNA show a high degree of specificity and sensitivity when used to detect DeMeth and Meth DNA in primary murine islets and βTC3 cells. Assay coefficient of variation for DMI of all samples excluding MS1 (which showed very low hypomethylated DNA) was calculated at 11.32 ± 2.68%.

### Amylin expression in the islet and demethylated amylin cfDNA are detected at the time of T1D in NOD mice

**Fig 3:**
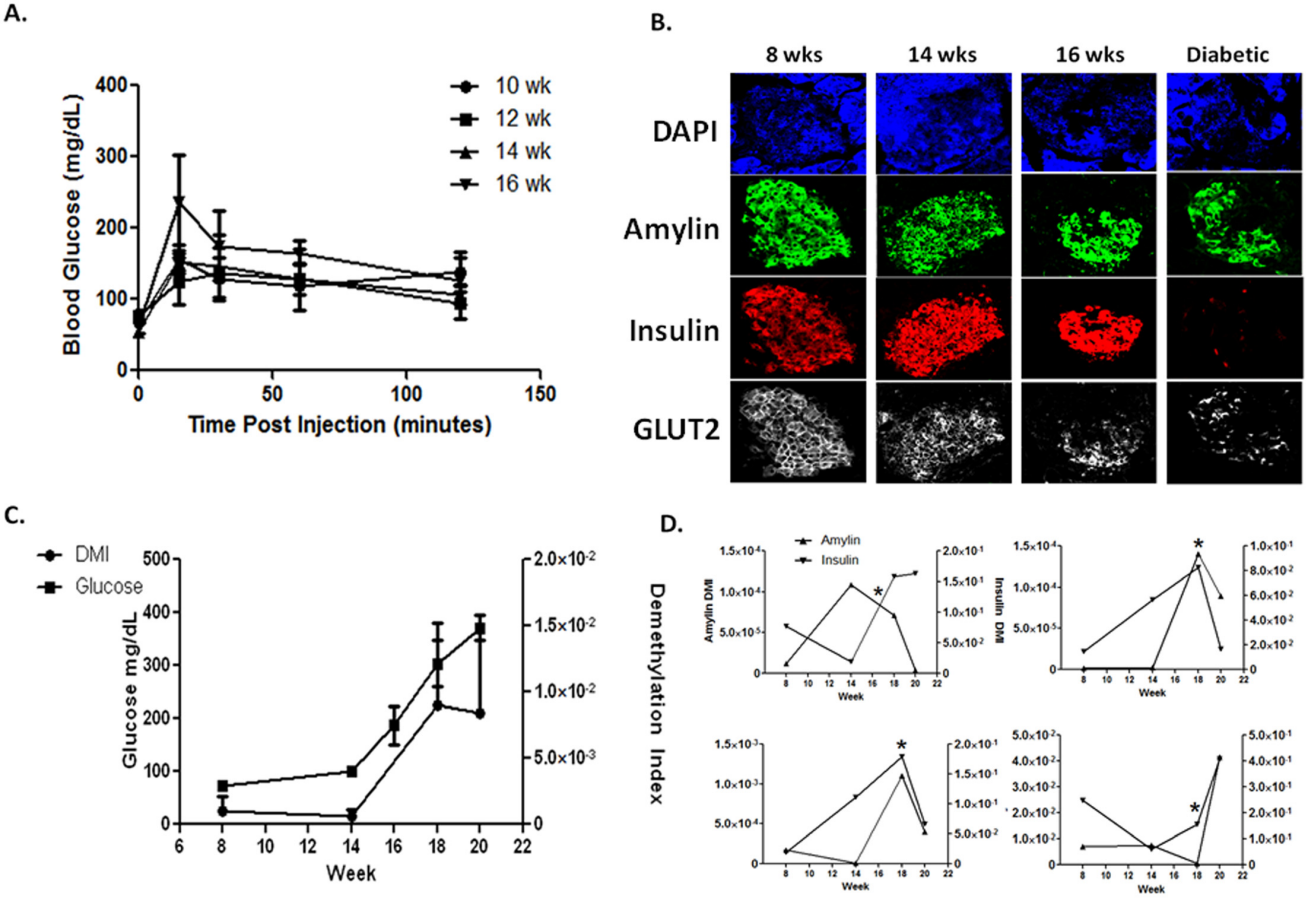
Demethylated amylin DNA is increased in the blood of pre-diabetic NOD mice during T1D progression. 8 wk old female NOD mice were housed in SPF conditions and monitored for 12 weeks for the development of diabetes. Blood from each animal was collected on wk 8, 14, 18 and 20. ***A*.** IPGTT values of pre-diabetic NOD mice at various ages over 120 minutes. ***B*.** Immunofluorescence staining of representative islets from NOD mice at various ages. Blue- DAPI. Green- Amylin. Red- Insulin. White- GLUT2. Note the appearance of insulin^-^amylin^+^GLUT2^+^ β-cells in islets from diabetic NOD mice ***C*.** Aggregate DMI and glucose values in NOD mice collected over 12 weeks (n = 14). ***D*.** Representative data from four individual NOD mice. DNA concentration in the serum was measured using picogreen. DMI was calculated on bisulfite treated serum-derived DNA. Variability in disease onset and β-cell DNA is characteristic of the spontaneous nature of T1D in the NOD mouse model.

We have previously shown that DeMeth insulin cfDNA levels are increased during the natural progression of T1D in the NOD mouse and are reduced following the development of hyperglycemia, demonstrating the utility of DeMeth insulin DNA as a biomarker of insulin-expressing β-cell death in prediabetes [8]. Here, 8 wk to 20 wk old NOD mice were followed for the development of hyperglycemia. In contrast with our previous reports, cheek pouch bleeding replaced heart puncture for blood collection, thereby providing a kinetic view of β-cell death in the same animal. IPGTT analysis showed a gradual deterioration in glucose tolerance at 16 wks of age (Fig 3A). IF analysis of insulin and amylin expression showed marked reduction in insulin expression at 16 wks. However, amylin expression remained relatively stable and amylin+/insulin- islets were observed throughout the pancreas well after diabetes was established (Fig 3B). These cells stained positive for GLUT2 confirming their β-cell phenotype (Fig 3B). This surprising finding suggests that a subset of amylin-expressing β-cells may persist following the development of hyperglycemia, which may otherwise remain undetected by insulin staining. Analysis of DMI values in NOD mice of different age showed an increase in β-cell death during diabetes progression (Fig 3C), with DeMeth amylin cfDNA peaking at the time of disease presentation. Analysis of insulin and amylin DMIs in individual NOD mice revealed a high degree of variability in DMI values prior to or during the presentation of hyperglycemia (Fig 3D). All in all, insulin and amylin DMI levels were discordant during the period of prediabetes but tended to follow a similar pattern at the time of diabetes presentation, suggesting that the two biomarkers may present two independent measurements of different β-cell subsets in these mice. Taken together, these results identify a β-cell population which remains amylin positive while losing insulin expression and demonstrate the ability of methylation-specific primers to detect an increase in amylin cfDNA at the time of disease presentation in the NOD model of T1D.

### Amylin DNA is demethylated in both primary human islets and enriched human β-cells and can be detected by methylation-specific primers

**Fig 4:**
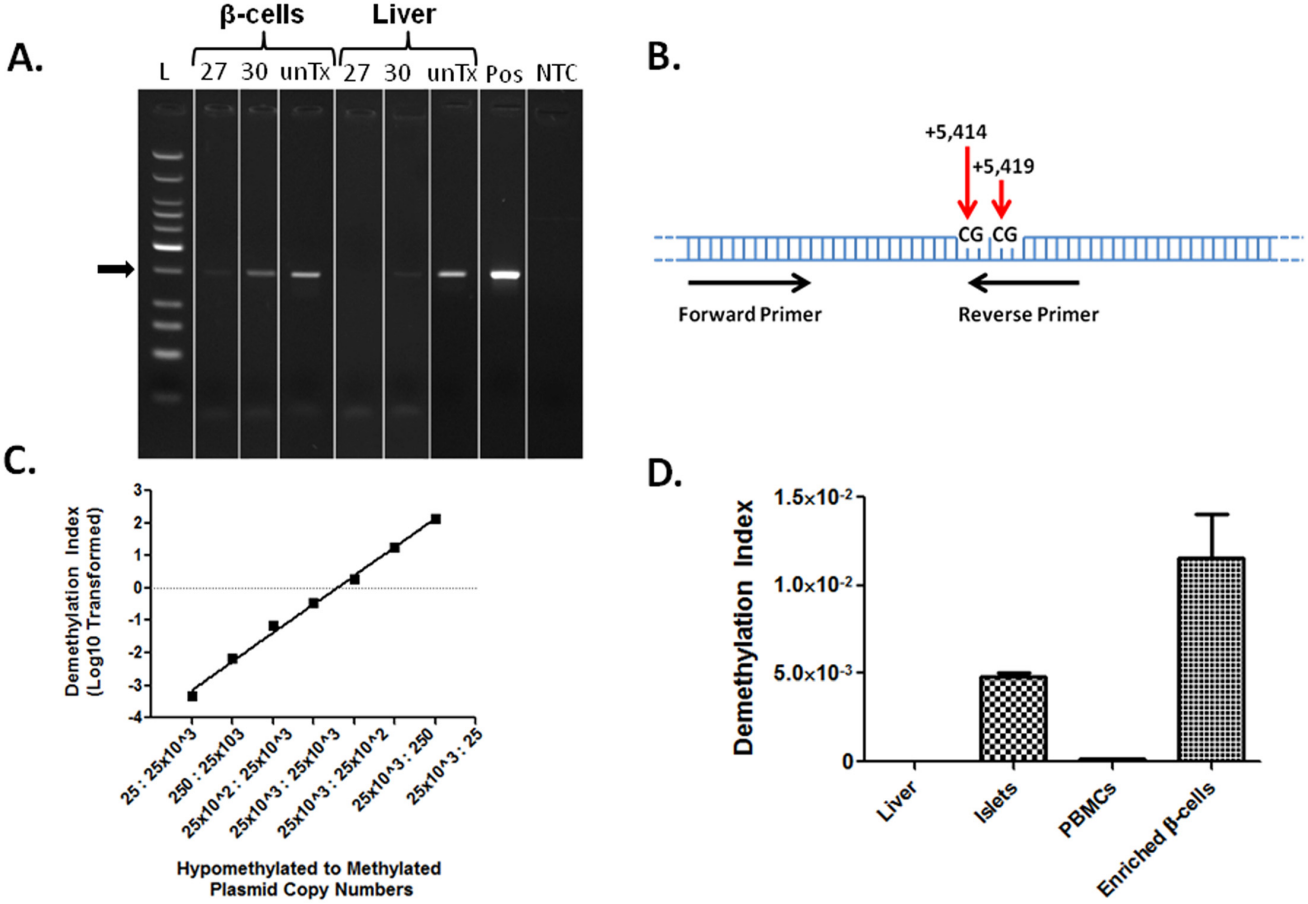
Methylation-specific primers show a high degree of specificity and sensitivity and detect demethylated DNA in primary human islets and enriched human β-cells. ***A*.** Methylation sensitive DNA digestion was performed on magnetically enriched β-cells and liver DNA using the McrBC enzyme. Digested DNA was subjected to semi-quantitative PCR (cycles 27 and 30) and run on agarose gel. unTx- untreated DNA receiving all reaction components except McrBC enzyme. Pos- positive control using cloned native amylin DNA. NTC- no template control. ***B*.** Schematic depiction of differentially methylated CpG dinucleotides in the human amylin coding region used for the design of methylation-specific primers. ***C*.** Human methylation-specific primers were tested over a wide range of copy numbers for DeMeth and Meth DNA (25 to 25x10^3^) to determine assay sensitivity and specificity using cloned DNA sequences. (R^2^ = 0.9930, p<0.0001). ***D*.** qPCR reaction using methylation-sensitive primers on bisulfite treated DNA from liver, purified human islets, peripheral blood mononuclear cells (PBMCs) and magnetic beads enriched human β-cells. Islet fractions from three individual donors were used. Data represents DMI values from single donor and consists of two independent repeats.

The region used for the construction of methylation-specific amylin primers in the mouse is conserved in the human amylin gene. Analysis of DNA methylation by the methylation-sensitive restriction enzyme, McrBC, revealed the presence of demethylated amylin DNA in magnetic bead-enriched β-cell fractions when compared with liver fractions (Fig 4A). The presence of differentially methylated CpGs in the human amylin gene allowed for the design of methylation-specific primers (Fig 4B and Table 3). Plasmid containing synthetic DNA representing human Meth or DeMeth amylin sequences were cloned and validated by sequencing, mixed at variable copy numbers of 25, 250, 25x10^2^ and 25x10^3^ copies over a six logarithmic concentration range, and analyzed by qPCR. qPCR analysis of DeMeth amylin showed a high degree of positive correlation between PCR signal and the number of DeMeth amylin DNA even when diluted at 1:1000 ratio in Meth amylin DNA (Fig 4C, R^2^ = 0.9930, p<0.0001). To determine whether methylation-specific primers were capable of detecting DeMeth β-cell DNA, DNA from liver, islet and magnetically-enriched β-cells was isolated and analyzed by qPCR. DMI values of primary human islets were ~590 fold higher than liver, while enriched β-cells were ~1,440 fold higher than liver (Fig 4D). This increase was consistent with amylin mRNA expression in β-cells (data not shown). Similarly to mouse, DMI values of DNA from peripheral blood mononuclear cells (PBMCs) were considerably lower than enriched β-cells (Fig 4D). Amylin gene methylation stability was tested by exposing the EndoC-βH1 human insulinoma cells [34] to streptozotocin for 24 and 48 hrs, showing steady DMI values between untreated and STZ-treated cells (S2 Fig). Taken together, methylation-specific primers for genomic amylin DNA show good assay sensitivity/specificity when tested using artificial DNA and primary human tissues. The overall increase in DMI in primary human islets and enriched β-cells suggests that methylation-specific primers may be used to detect β-cell-derived DeMeth amylin DNA in peripheral blood samples.

### DeMeth amylin cfDNA is increased in plasma of recent onset T1D patients

**Fig 5:**
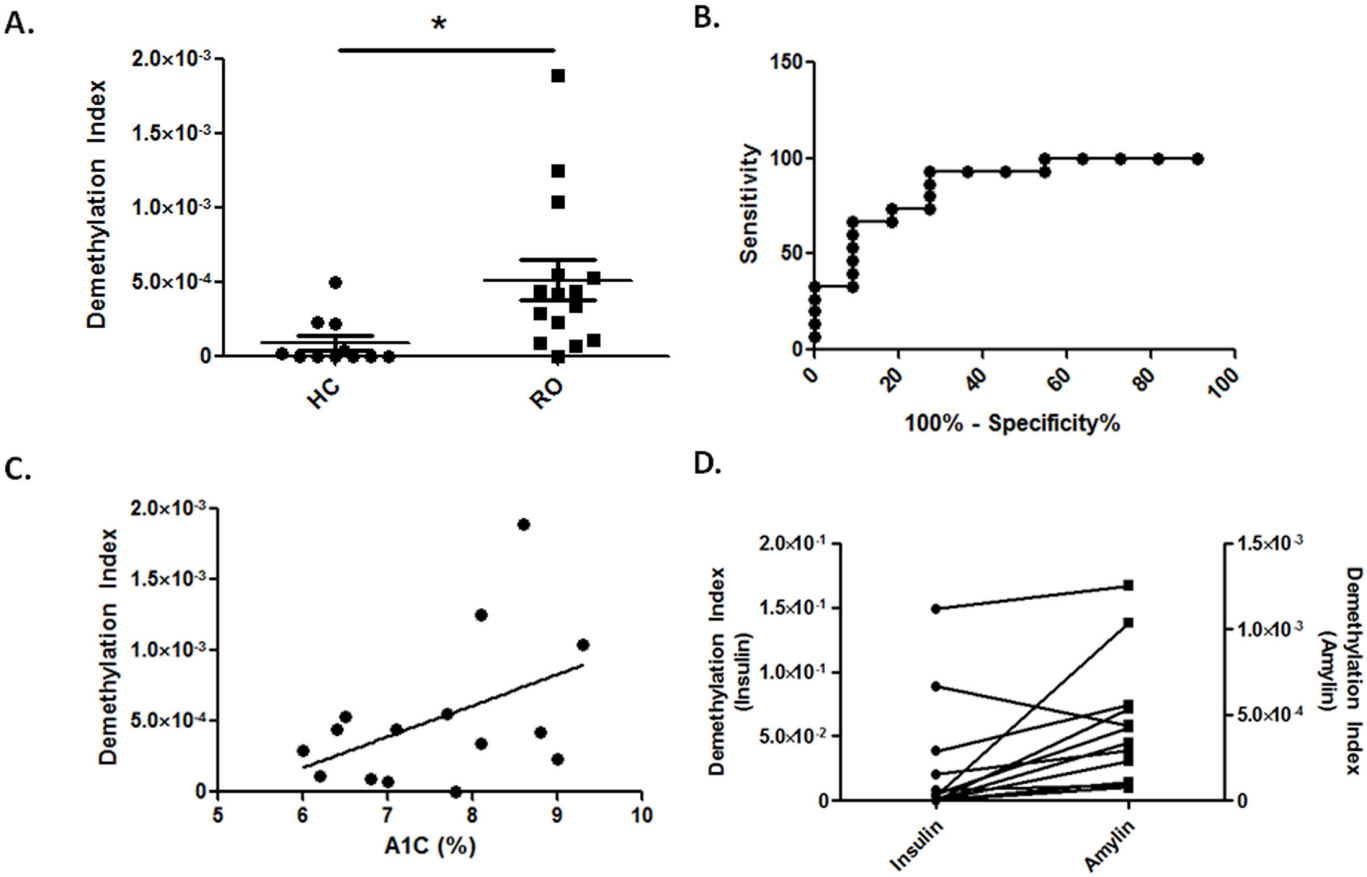
Methylation specific primers show increased demethylated amylin DNA in the blood of patients with recent onset T1D. ***A*.** DMI values for healthy control (HC, closed circles) and recent onset T1D patients (RO, closed squares), p<0.015. ***B*.** ROC analysis of patient data. AUC = 0.866, with 95% confidence interval 0.72 to 1.01, p<0.0017. ***C*.** Correlation analysis between HbA1c and DMI values in RO patients. ***D*.** Data presentation of insulin/amylin DMI per RO patient. Pearson’s r = 0.63, p<0.028.

Amylin cfDNA levels were increased at the time of disease onset and persisted in diabetic NOD mice, prompting us to test whether methylation-specific human amylin primers can detect amylin cfDNA in plasma samples from RO T1D patients and age-matched unrelated HC collected at the Children’s Hospital of Wisconsin. Plasma samples were processed and cfDNA extracted, bisulfite-converted, and subjected to first step PCR as described in Materials and Methods. Amplicons were gel-purified to remove impurities and qPCR was done using methylation-specific primers. qPCR analysis revealed a statistically significant increase in DeMeth DNA in the RO T1D group (Fig 5A, p<0.015) when compared with HC individuals. ROC analysis of samples showed an AUC of 0.866 with 95% confidence interval of 0.72–1.01. This analysis reached statistical significance (Fig 5B, p<0.0017). Correlation analysis between DMI values and HbA1c at the time of sampling showed a modest positive correlation between impaired glycemic control and DMI values (Fig 5C, R = 0.458, p<0.083). In our previous reports we have shown that insulin can be used as a biomarker of β-cell death in T1D [8]. Therefore, we examined DMI levels of insulin in the same patient cohort. Analysis of amylin and insulin signals showed that the increased levels of amylin DMI were associated with increased insulin DMI values (Fig 5D, Pearson’s r = 0.63, p<0.028).

## Discussion

In this report we identified the presence of differentially methylated CpG dinucleotides in the coding region of the amylin gene in murine and human islets and β-cells. These unique patterns can be used as a biomarker of β-cell loss in the NOD mouse model of T1D and in patients with RO T1D. Since amylin protein expression persists in murine islets even after insulin expression has been lost, this new assay can serve as a biomarker of β-cell loss in addition to our previously described insulin biomarker, thereby providing a dual-gene approach for evaluating β-cell loss in T1D.

Our group and others have previously demonstrated the utility of differentially methylated insulin DNA as a biomarker of β-cell loss in T1D [8–10], as insulin is uniquely expressed in these cells. Similarly, amylin is highly expressed in the islet by β-cells and secreted together with insulin [15–17]. Our analysis of methylation in the amylin gene coding region has revealed several unique demethylated patterns in β-cells when compared with other murine and human tissues. Similar methylation patterns were also found in insulinoma cells, suggesting that DNA methylation may play a role in the control of amylin expression in β-cells. Additional studies examining the methylation status of the amylin promoter will be needed to establish the role of methylation in the control of amylin gene expression.

The presence of β-cell-specific methylation patterns in the amylin gene provided an opportunity to develop methylation-specific primers capable of distinguishing between β-cell-derived DeMeth DNA and Meth DNA from all other tissues. Meth and DeMeth amylin DNA-sensitive primers for both murine and human amylin sequence showed a high degree of specificity and sensitivity when tested using cloned Meth and DeMeth amylin DNA. This was evident by the ability of the assay to maintain a linear pattern throughout a wide range of DNA concentrations, and an ability to detect as little as 25 copies of DeMeth DNA (equivalent to 12.5 β-cells) even when diluted in 25,000 copies of Meth DNA (equivalent to 12,500 non-β-cells). Analysis of DNA from different tissues showed similar results, with human and mouse islets and enriched β-cells yielding higher DMI values when compared with other tissues.

Methylation-specific primers provided a tool for detecting DeMeth DNA in serum of NOD mice. The loss of β-cells in this model has been extensively studied [37], and our data showing a deterioration of both glucose tolerance and insulin staining in the islet support these findings. Immunofluorescence staining of islets from prediabetic and diabetic mice revealed a disconnect between insulin and amylin protein expression and and were supported by an increase in amylin mRNA expression in human insulinomas following exposure to high dose streptozotocin (S2 Fig). These novel findings in the islet are supported by a previous report demonstrating similar levels of amylin protein in the blood of prediabetic and diabetic NOD mice [38]. The loss of insulin but not amylin expression in this subset of β-cells may make these cells invisible to a biomarker assay that relies solely on the detection of insulin cfDNA. Indeed, DeMeth amylin DNA levels were increased in prediabetic NOD mice reaching a peak at disease presentation, and were not correlated with DeMeth insulin levels in the serum. Our ability to obtain sufficient cfDNA by periodic cheek pouch bleeding in the same mouse is an important advancement over previous reports that required heart puncture or terminal bleeding of the mice. This approach allows for a longitudinal view of diabetes progression and a measure of β-cell loss over time.

The human amylin coding region shares a high degree of sequence homology with the mouse gene. Homologous sequences in human amylin DNA demonstrated differential methylation between β-cells and liver by methylation-sensitive enzymatic DNA digestion. Methylation-specific amylin primers showed a high degree of specificity and sensitivity to artificially DeMeth DNA as well as DNA from primary human islets and enriched β-cells. When used to test the levels of DeMeth amylin DNA in human subjects, these primers demonstrated a statistically significant increase in DeMeth amylin cfDNA in RO T1D patients when compared with unrelated HC, with good assay specificity and sensitivity by ROC analysis. Moreover, the mild correlation between DMI and HbA1c values may suggest that diabetes severity due to poor metabolic control and immune dysregulation may contribute to β-cell loss. Finally, although DMI values of amylin and insulin cfDNA were in overall agreement in RO T1D patients, amylin cfDNA levels showed a stronger increase in amylin signal than insulin, suggesting that amylin expression may persist in the islets of diabetic patients in a similar fashion to diabetic NOD mice. This hypothesis is supported by previous reports showing a deviation in the concentration of c-peptide/insulin and amylin in the plasma of T1D patients [39]. An alternative explanation may relate to the fact that the levels of cfDNA in the blood are low, thereby allowing for the detection of amylin but not insulin in some serum samples and vice versa. In any event, the combination of both insulin and amylin DMI offers a unique opportunity for a dual gene approach to measure β-cell loss, which would otherwise remain undetected by the insulin biomarker assay. This dual gene assay can enhance assay validity and reliability by expanding assay measurement to more than a single gene for β-cell loss detection.

Here we report differential methylation of the amylin gene in the islet and in enriched β-cells. This differential methylation of amylin in β-cells provides an opportunity to detect the presence of β-cell-derived DeMeth amylin cfDNA by using methylation-specific primers. The identification of amylin^+^insulin^-^ β-cells highlights the importance of using amylin cfDNA as an additional biomarker of β-cell death in RO T1D patients in conjunction with our previously reported insulin gene.

## Supporting Information

S1 Fig DMI value of magnetically enriched (β cell (+)) or depleted (β cell (-)) β cell fraction. Primary β-cell were enriched using magnetic beads as described in Materials and Methods. DMI values showed a significant enrichment of demethylated insulin DNA in purified β-cells.
(PPTX)

S2 Fig The effects of acute STZ exposure on amylin gene expression and DMI values. Endo-C human insulinoma cells (obtained from the laboratory of Dr. R. Scharfmann, CRICM, Paris, France) were exposed to high streptozotocin (15 mM) for a period of 24 and 48 hrs. ***A*.** Phase contrast light microscopy showed a gradual loss of Endo-C cells integrity. ***B*.** Amylin real time analysis showed an increase in amylin gene expression; however this increase did not reach statistical significance. ***C*.** DMI values of genomic DNA pointed to stability in the methylation status of CpG pairs +5,414 and +5,419 (listed in Fig 4B).
(PPTX)
