# Supplementary figures and images for: Circulating Differentially Methylated Amylin DNA as a Biomarker of β-Cell Loss in Type 1 Diabetes
Source: PLoS One. 2016 Apr 25;11(4):e0152662. doi: 10.1371/journal.pone.0152662 (PMC4844136; doi:10.1371/journal.pone.0152662)

## Slide 1
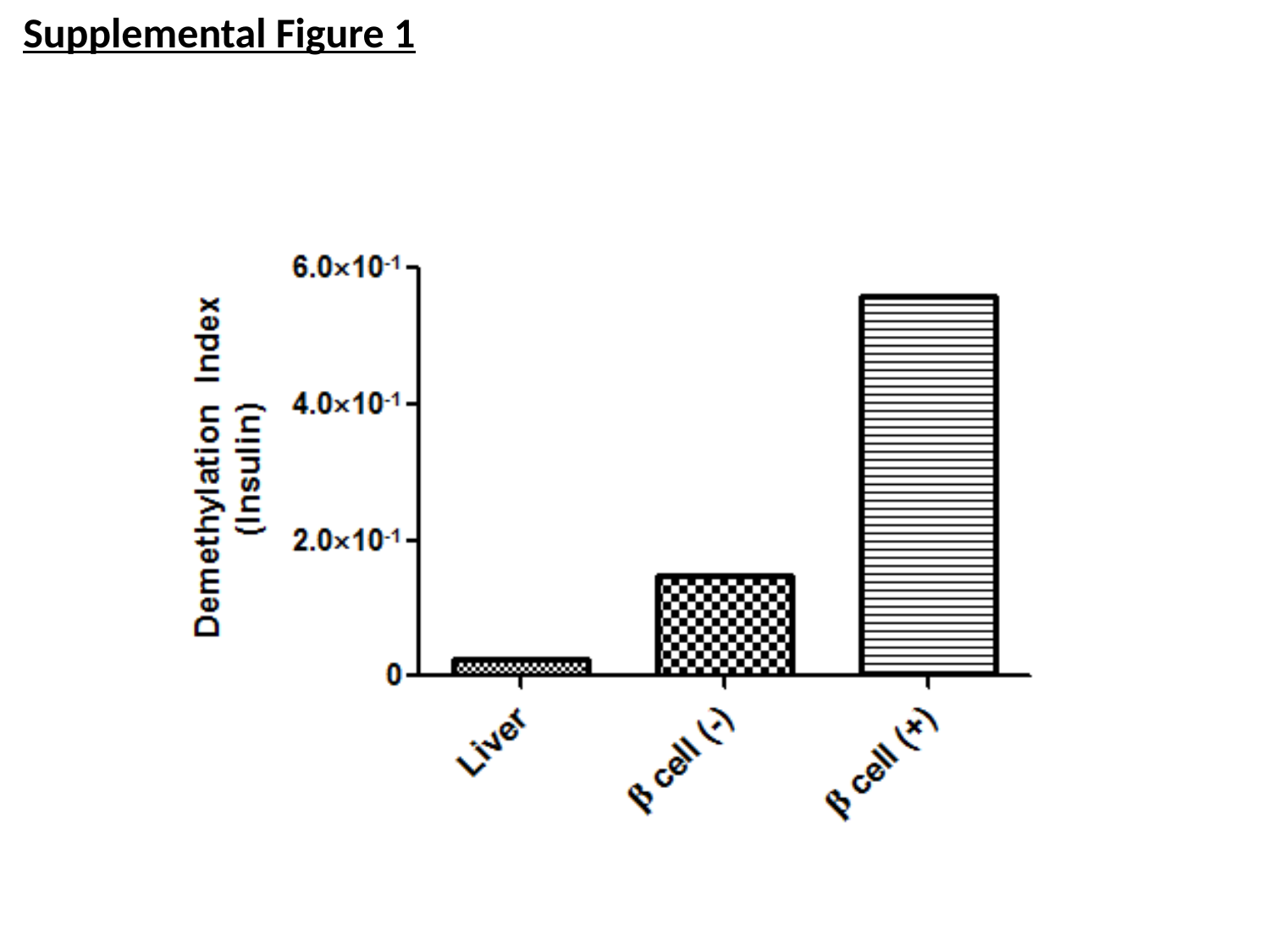

Supplemental Figure 1

Supplement: S1 Fig — Primary β-cell were enriched using magnetic beads as described in Materials and Methods. DMI values showed a significant enrichment of demethylated insulin DNA in purified β-cells. (PPTX) [file pone.0152662.s001.pptx]
