# Supplementary material for: Circulating Differentially Methylated Amylin DNA as a Biomarker of β-Cell Loss in Type 1 Diabetes
Source: PLoS One. 2016 Apr 25;11(4):e0152662. doi: 10.1371/journal.pone.0152662 (PMC4844136; doi:10.1371/journal.pone.0152662)
Supplement: S2 Fig — Endo-C human insulinoma cells (obtained from the laboratory of Dr. R. Scharfmann, CRICM, Paris, France) were exposed to high streptozotocin (15 mM) for a period of 24 and 48 hrs. A. Phase contrast light microscopy showed a gradual loss of Endo-C cells integrity. B. Amylin real time analysis showed an increase in amylin gene expression; however this increase did not reach statistical significance. C. DMI values of genomic DNA pointed to stability in the methylation status of CpG pairs +5,414 and +5,419 (listed in Fig 4B). (PPTX) [file pone.0152662.s002.pptx]

## Slide 1
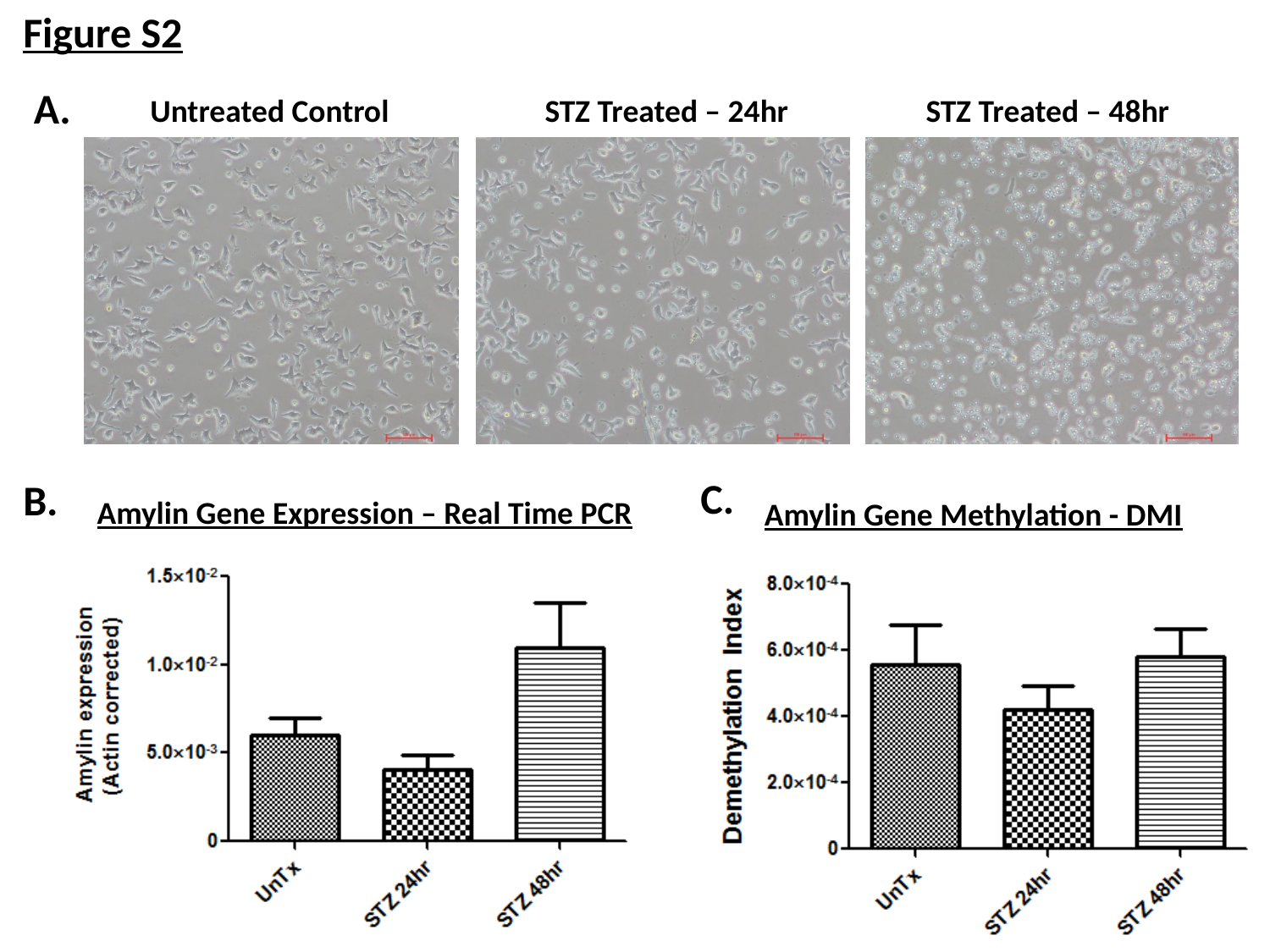

Figure S2
A.
Untreated Control
STZ Treated – 24hr
STZ Treated – 48hr
C.
B.
Amylin Gene Expression – Real Time PCR
Amylin Gene Methylation - DMI
